# Efficacy of a Mindfulness-Based Intervention in Ameliorating Inattentional Blindness Amongst Young Neurosurgeons: A Prospective, Controlled Pilot Study

**DOI:** 10.3389/fsurg.2022.916228

**Published:** 2022-05-06

**Authors:** Anand S. Pandit, Melissa de Gouveia, Hugo Layard Horsfall, Arisa Reka, Hani J. Marcus

**Affiliations:** ^1^ Victor Horsley Department of Neurosurgery, National Hospital for Neurology and Neurosurgery, London, United Kingdom; ^2^ Wellcome/EPSRC Centre for Interventional and Surgical Sciences, University College London, London, United Kingdom; ^3^ UCL Medical School, London, United Kingdom

**Keywords:** inattention blindness, cognitive load, safety, surgical training, education, cognitive bias

## Abstract

**Background:**

Human factors are increasingly being recognised as vital components of safe surgical care. One such human cognitive factor: inattention blindness (IB), describes the inability to perceive objects despite being visible, typically when one’s attention is focused on another task. This may contribute toward operative ‘never-events’ such as retained foreign objects and wrong-site surgery.

**Methods:**

An 8-week, mindfulness-based intervention (MBI) programme, adapted for surgeons, was delivered virtually. Neurosurgical trainees and recent staff-appointees who completed the MBI were compared against a control group, matched in age, sex and grade. Attention and IB were tested using two operative videos. In each, participants were first instructed to focus on a specific part of the procedure and assessed (attention), then questioned on a separate but easily visible aspect within the operative field (inattention). If a participant were ‘inattentionally blind’ they would miss significant events occurring outside of their main focus. Median absolute error (MAE) scores were calculated for both attention and inattention. A generalised linear model was fitted for each, to determine the independent effect of mindfulness intervention on MAE.

**Results:**

Thirteen neurosurgeons completed the mindfulness training (age, 30 years [range 27–35]; female:male, 5:8), compared to 15 neurosurgeons in the control group (age, 30 years [27–42]; female:male, 6:9). There were no significant demographic differences between groups. MBI participants demonstrated no significant differences on attention tasks as compared to controls (*t *= −1.50, *p* = 0.14). For inattention tasks, neurosurgeons who completed the MBI had significantly less errors (*t *= −2.47, *p* = 0.02), after adjusting for participant level and video differences versus controls. We found that both groups significantly improved their inattention error rate between videos (*t *= −11.37, *p* < 0.0001). In spite of this, MBI participants still significantly outperformed controls in inattention MAE in the second video following post-hoc analysis (MWU = 137.5, *p* = 0.05).

**Discussion:**

Neurosurgeons who underwent an eight-week MBI had significantly reduced inattention blindness errors as compared to controls, suggesting mindfulness as a potential tool to increase vigilance and prevent operative mistakes. Our findings cautiously support further mindfulness evaluation and the implementation of these techniques within the neurosurgical training curriculum.

## Introduction

Patient safety has been at the centre of healthcare for millennia, summarised by Hippocrates’ (470–360 BC) as “first, do no harm”. This remains a fundamental mantra for modern medicine. Errors in neurosurgery, compared with other surgical settings, can be particularly catastrophic and can result in significant events for the patient, surgeon, and institution (1, 2). Much work has focused on improving patient safety across all surgical specialities, and international initiatives like the WHO Surgical Safety Checklist, have driven a paradigm shift in protocol and culture. Despite this increased awareness of safety, errors continue to routinely occur in surgical care (3). This heralds a systems-approach to surgical safety and the need to consider human factors in the delivery of surgical services.

One example of a human factor in surgery is inattention blindness (IB) – the inability to perceive objects that are visible, when one’s attention is focused on another task (4, 5). Failure to recognise and act upon events during surgery can lead to serious clinical incidents, including so-called “never-events”, such as retained foreign objects and wrong site surgery (3). These preventable incidents can lead to significant, patient harm, particularly in neurosurgery (2) and often have no relation to other features of safety (6). Contributing to inattention blindness is the difficulty in sustaining a vigilant state for long periods of time, which itself is highly cognitively demanding (7).

Mindfulness is the capacity to monitor sensory and perceptual stimuli and experiences, moment-by-moment in a non-judgmental manner (8). By packaging this construct within a group-based intervention, it has been successfully implemented for patients (9) and has since been trialled as a technique that can reduce burnout among residents, in particular, surgeons (10, 11). Even brief formats of this intervention have recently been shown to improve performance-related factors among surgeons and peri-operative staff in the operating theatre (12). It is likely, at least in part, that the benefits associated with mindfulness in functional performance are linked to improvements in attention, emotional regulation and decision-making which the technique specifically entrains (13). Further, it is postulated that directed mindfulness training can foster greater attention to, and awareness toward ongoing sensory and perceptual stimuli and experiences (13). Indeed, the situational alertness established as a result of mindfulness training may aid in ameliorating inattention blindness.

To that end, we aimed to determine if a mindfulness-based intervention (MBI) reduced inattention blindness among young neurosurgeons, namely, those in training or who had recently been appointed as staff. That this particular group tend to incur more operative complications (14), adopt differing learning mindsets (15) and use different training resources (16) as compared to experienced consultants marks their unique characteristics and potential for improvement with mindfulness training.

Our primary hypothesis was that participants who completed the MBI would demonstrate reduced inattention blindness in a situational operative task as compared to matched controls. We also tested the hypothesis that attention would improve among participants in the interventional group.

## Methods

The study was designed and reported in accordance with STROBE (17) and CONSORT guidelines (18) with adaptation for non-randomised pilot studies (19).

### Ethical Approval

The use of data obtained from the course was approved by our academic Institutional Review Board (17019/001) and all subjects gave informed consent.

### Participants

Participants were recruited through newsletters from U.K. and European neurosurgical societies, social media groups, and word of mouth. All participants self-referred, and after expressing interest, they were given more information about the study before giving written consent. Surgeons were not obliged to participate in the research study in order to receive mindfulness training. A matched cohort of control participants were recruited from our institution based on surgical grade, age and gender. Individuals who had previously completed a formal mindfulness training programme were excluded from the study. Also excluded were participants not presently working in neurosurgery or attendings who had been in post for more than a year.

### Mindfulness Intervention

From October to December 2020, an 8-week virtual MBI programme was delivered by a mindfulness instructor (a physician with 5 years of teaching experience and approximately 5000 h of personal practice time). Weekly 90-minute sessions had specific themes and exercises (see Supplementary Methods). Participants were invited to voluntarily practice in their own time and utilise the online learning platform provided. The intervention was based on a course tailored specifically for healthcare professionals that incorporated core concepts of mindfulness and (self-)compassion training (20). The intervention was designed to promote emotional intelligence competencies in clinical environments including self-awareness, self-management, and social awareness. Practical and conceptual differences between our course and traditional mindfulness therapies (9) such as mindfulness-based stress reduction (MBSR) are outlined in the Supplementary Material, however the course was similar in concept and design to established, evidence-based surgically directed courses such as enhanced stress resilience training (ESRT) (21–23).

### Attention and Inattention Tasks

Within two weeks of completing the MBI, both intervention and control groups were assessed virtually using a task designed to recruit attention and provoke inattention, in an environment and time convenient to each participant. This was adapted from previous work done by Dixon et al. (24), and follows a similar format to the seminal ‘Gorilla’ study by Simons and Chabris which examined inattentional blindness for complex objects and events in dynamic scenes via a video medium (25). The tasks were calibrated using a seperate, interventional-naive group of neurosurgeons (ranging from intern to attending). This was to ensure content and face validity, an appropriate difficulty level and that the tasks were clinically-relevant, specifically clarifying situations in which inattention blindness may contribute to significant patient harm (e.g. where a swab may be retained).

**Figure 1:**
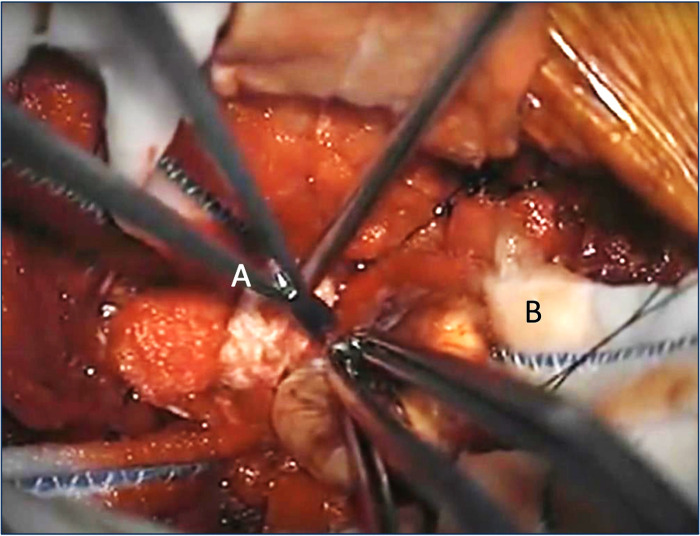
Example screenshot from a spinal surgery operative video, where a nerve sheath tumour is being excised. Participants in the study were asked to focus on and count how many times the scissors (**A**) were used to cut in the attention task. They were then asked to identify the number of cottonoid patties (**B**), outside of their focus, left in the surgical site at the end of the video in the inattention task. Absolute error scores were calculated by taking the difference between the stated and actual count. (Full video available at https://www.youtube.com/watch?v=dGeSd5hU1tw, Prof. Ali Bydon, Johns Hopkins Medicine, uploaded 18th July, 2012. Image reproduced here with permission from the author)

Each group was shown two 90 s operative videos: the first procedure familiar to neurosurgeons (spinal surgery) and the second unfamiliar and more cognitively demanding (splenectomy). In each video, participants were asked to focus on a part of the procedure (e.g., counting the number of times an instrument was used, i.e. *attention*) and assessed, then questioned on an aspect thought to be out of the scope of the scenario but present in the operative field (e.g., counting the number of swabs left in the surgical site i.e. *inattention*) [Figure 1]. Participants were intentionally not informed in advance that the task was designed to test inattention blindness nor revealed that factors other than the area of focus would be assessed. A median absolute error (MAE) score was calculated for both attention and inattention aspects of the video corresponding to the difference between perceived and actual counts. If a participant were ‘inattentionally blind’ they would miss significant events occurring outside of their main focus and have a higher error score.

### Data Analysis

Given the nature of this pilot study, an upper sample threshold of *n* = 25 was derived following consultation with the mindfulness instructor regarding the typical class size limit. The minimum sample size was based on a practical limit of participant recruitment. Participants who failed to complete a minimum of five sessions to receive course certification were excluded from further statistical analysis.

All statistical tests and data visualisation were performed using Python (3.8.6) and RStudio (1.2.1335). Tests for normality were conducted using the Kolmogorov-Smirnov test, and by visually inspecting the distribution and q-q plots of the data and residuals respectively. Given that the procedure video, participant differences and intervention may each have an effect on a surgeon’s error score, a generalised linear model was fitted with these predictors and the scaled MAE as the dependent variable for attention and inattention seperately. Interactions between other variables and designation of ‘random’ or ‘fixed’ effects were permuted to find the model and distribution with the best fit, namely that one with the lowest Akaike Information Criterion (Supplementary Results).

A *p*-value of <0.05 was considered significant throughout. If the omnibus test was significant, a post-hoc independent t-test or Mann-Whitney U test was employed to assess for specific differences between intervention and control groups. If differences were found between first and second operative videos, a paired t-test or Wilcoxon-Signed-Rank test was then performed.

## Results

### Demographics

Twenty-one neurosurgeons were recruited to undertake the mindfulness course. Five were ineligible for further analysis due to insufficient sessions being attended and 3 did not complete the post-interventional attention assessment. Thirteen neurosurgeons completed the mindfulness training (median = 7 out of 8 sessions completed) and were eligible for further analysis (age, 30 years [range 27–35]; female:male, 5:8), compared to 15 neurosurgeons in the control group (age, 30 years [27–42]; female:male, 6:9). There were no significant demographic differences between intervention and control groups demonstrating adequate matching (Table 1).

**Table 1:** Demographic characteristics of participants.

	MBI	Controls	*p*
*n*	13	15	–
Median age (range)	30 (27–35)	30 (27–42)	0.87 (MWU)
Sex (F)	5	6	0.92 (χ^2^)
Grade (SHO/SpR/Consultant)	8/5/0	11/3/1	0.40 (FE)

*SHO: senior house officer - equivalent to junior resident; SpR: specialist registrar - equivalent to senior resident; MBI: mindfulness-based intervention; MWU: Mann-Whitney U test; FE: Fisher-Exact test.*

### Attention

MBI participants demonstrated no differences on attention tasks, compared to controls (*t* = −1.50, *p* = 0.14), although a trending difference was found for the first video (MWU, *p* = 0.08) with controls having higher error scores.

**Figure 2:**
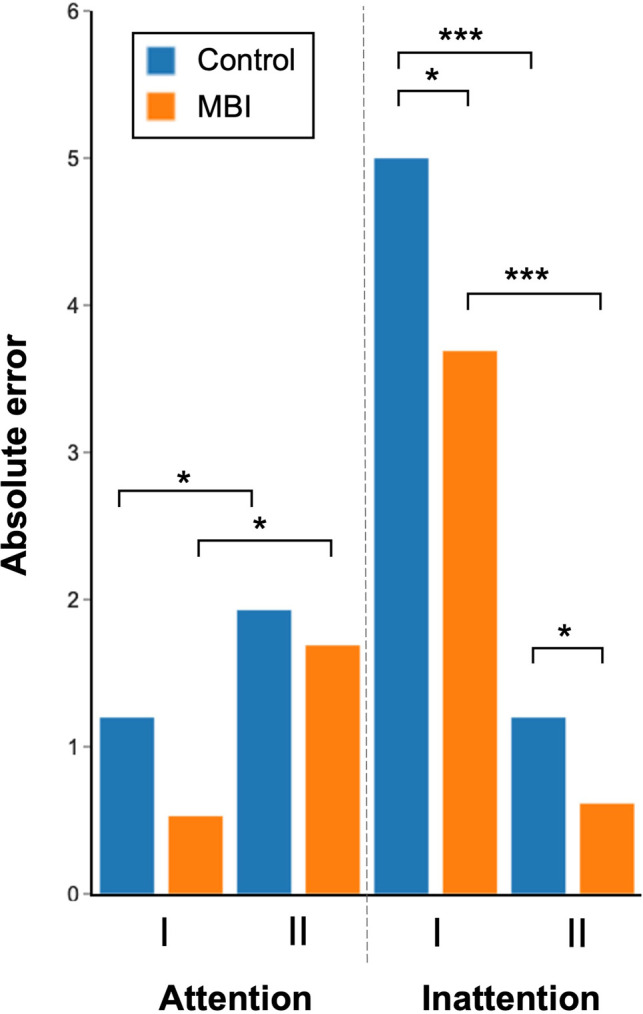
Absolute error scores for attention and inattention tasks for the first (I) and second (II) operative videos. For visualisation purposes the mean absolute error for each group is demonstrated as the height of each bar. (*p ≤ 0.05, ***p < 0.001).

Both groups did demonstrate significant differences between operative videos (*t* = 3.84, *p* < 0.0001). Here, across all participants accounting for their differences and intervention status, error scores increased by 0.92 in the second operative video as compared to the first. On post-hoc analysis, the mindfulness group deteriorated between the first operative video (MAE = 0, range = 0–2) and second (MAE = 2, range = 0–3, Wilcoxon-Rank-Sum = 2.5, *p* = 0.02). Similarly, the control group’s attention errors significantly increased between the first (MAE = 0, range = 0–2) and second operative videos (MAE = 1, range = 0–3, Wilcoxon-Rank-Sum = 2.5, *p* = 0.03) [Figure 2].

### Inattention

Inattention scores significantly differed between the MBI group and controls (*t* = −2.47, *p* = 0.02). Here, after accounting both for differences between individuals and videos, by completing the MBI, a participant’s error score improved by 0.36. Accordingly, there was a significant difference in error metrics between the MBI group (video 1, MAE = 4, range = 0–6; video 2, MAE = 0, range = 0–3) and controls (video 1, MAE = 5, range = 1–6, MWU = 147.5, *p* = 0.02; video 2, MAE = 1, range = 0–3, MWU = 137.5, *p* = 0.05). Inattention scores also differed significantly between the first and second operative videos for both groups (*t* = −11.37, *p* < 0.0001), where inattention errors were reduced by −1.64 when watching the second video [Figure 2].

A repeat analysis was also performed, excluding the single consultant from the control group. This resulted in no changes to the significance or direction of the aforementioned results.

## Discussion

In this brief prospective, proof-of-principle study, young neurosurgeons undertaking formal mindfulness training had significantly greater recognition of an unexpected distractor and reduced inattention-related error scores. After the first video was completed, both groups were now aware of the experiment’s motive and likely to be more vigilant for other unexpected distractors. Although control participants significantly improved their inattention error rate in the second video procedure, they were still outperformed by MBI participants who had significantly less inattention-related errors. This would suggest that mindfulness exerts an additional effect over the cognitive conspicuity (26) of previously unperceived factors in the operative field in the first video. Using a multivariate model, we found that amelioration of inattention blindness was found to be independently associated with mindfulness training after adjusting for individual and task-related differences.

In contrast, error-metrics related to attention were not significantly different between MBIs and controls in either operative video, although a trend existed for the first procedure. Both groups performed worse during the second procedure as compared to the first, likely, at least in part, because of the added complexity associated with an operation unfamiliar to most neurosurgeons. It is unclear why greater group-wise differences in attention were not observed, since attentional regulation forms a core component of mindfulness training (27). This may be due to high baseline levels of attention, already entrained in this actively working group.

Recent iteratively adapted and tailored MBIs have demonstrated improvements in psychomotor performance, executive function, and attenuated negative psychological states among clinicians (28), and specifically, surgical trainees (22). None, however, have examined the role of mindfulness in reducing harmful cognitive factors within the operating room.

Our findings are aligned with previous work suggesting that mindfulness increases awareness of unexpected stimuli (13). This may be mediated by a number of mechanisms including reductions in stress and cognitive load (10, 29) and increases of working memory (30), all of which may help ameliorate inattention blindness (31). Mindfulness training represents a versatile, low-cost solution to foster operative vigilance and reduce technical errors (32). Certainly, if a pragmatic solution to reduce intraoperative technical errors were available it would have dramatic societal impact. Gawande et al found that 66% of all in-hospital adverse events were found to be surgical in nature (33), most of which occurred in the operating theatre, and that 54% were found to be preventable. Furthermore, a lack of vigilance represents one of the main cognitive factors leading to surgical errors and patient complications (34, 35). While it is accepted that surgery is not-error free, surgical excellence represents the ability to anticipate and manage errors and problematic events during surgery (36). Thus, surgeons engaging in mindfulness practice might be better prepared to deal with intraoperative events and reduce harm to patients, through enhanced awareness of the surgical field.

It has been suggested that mindfulness cannot be taught explicitly, rather modelled by mentors and cultivated in learners (32) and that such learning occurs during observation and practice, and over time. Surgeons who undertook the brief, targeted virtual mindfulness intervention in this study demonstrated a greater recognition of unexpected distractors compared with controls. This suggests that if appropriately directed, surgeons can develop mindfulness as a procedurally-relevant tool in a relatively short time span. These findings lend support toward the integration of mindfulness training within institutional teaching programs, and more widely within the neurosurgical speciality curriculum (37) albeit from an angle of improving patient safety rather than personal resilience. Alongside their personal and professional benefits, it has been shown that mindfulness interventions are both acceptable and feasible in surgical departments where they are used, with no negative effect on surgical training (38). This makes a compelling case for including this intervention in the residency curricula, which are mandated by national accreditation organisations (39, 40).

The limitations of this study include the small, matched but not randomised, sample. We also acknowledge that ameliorating inattention blindness via mindfulness techniques in the heavily constrained, virtual environment may not directly or immediately translate to improved patient outcomes. All sessions and assessments were performed virtually due to the COVID-19 pandemic, potentially resulting in heterogeneous conditions for participants. For the MBI group, attending the weekly sessions were mandatory, whereas “offline” practice was voluntary and therefore difficult to quantify. The control group for comparison was not a waitlist or dummy group and therefore there may have been a selection bias owing to the endogenous differences in motivation amongst the mindfulness participants versus controls (22). The attention and inattention tasks used in this study were somewhat novel, and although some measures were taken to validate each scale, assessments of reliability would have been challenging. Although a test/retest reliability assessment would be an appropriate method for single outcome instruments (41), in this case the ‘trick’ of a hidden distractor would be apparent to participants taking the second test, meaning that scores would be poorly correlated.

## Conclusion

Neurosurgeons who underwent an eight-week MBI had significantly reduced inattention blindness errors as compared to controls, suggesting mindfulness as a potential tool to increase vigilance and prevent operative mistakes. In spite of the aforementioned limitations, we emphasise that this is the first study, to the best of our knowledge, which specifically evaluates the use of mindfulness in attenuating a procedural bias among clinicians, and represents one of few techniques available to improve surgical vigilance in the operating environment. Our findings cautiously support further mindfulness evaluation with larger, randomised cohorts of surgeons, and, if proven, the implementation of these techniques within the neurosurgical training curriculum.

## Data Availability

The raw data supporting the conclusions of this article will be made available upon reasonable request.
