# Supplementary material for: Efficacy of a Mindfulness-Based Intervention in Ameliorating Inattentional Blindness Amongst Young Neurosurgeons: A Prospective, Controlled Pilot Study
Source: Front Surg. 2022 May 6;9:916228. doi: 10.3389/fsurg.2022.916228 (PMC9122266; doi:10.3389/fsurg.2022.916228)
Supplement: Supplementary file 1 [file fsurg-09-916228_Table_1_v1.docx]

**Efficacy of a mindfulness-based intervention in ameliorating inattentional blindness amongst young neurosurgeons: a prospective, controlled pilot study**

## Supplementary Methods

### **Mindfulness intervention**

The overall course aim was to promote key emotional intelligence competencies in clinical environments: self-awareness, self-management, social awareness, and relationship management. Practical and conceptual differences between the Mindful Medics course and traditional mindfulness therapies such as MBSR are outlined:

(i) Audience: The Mindful Medics course was designed with healthcare professionals in mind and was adapted for surgeons through pre-course modifications made in collaboration with authors A.S.P, H.J.M and the course facilitator, by identifying typical day-to-day issues and stressors in clinical practice.

(ii) Class duration: sessions were 90 minutes and delivered outside of regular clinical hours to accommodate participants with demanding schedules.

(iii) Online learning: access was provided to additional information and exercises that would aid in the embedding of learning and maintenance of positive behavioural change.

(iv) Practices and context: whereas traditional courses typically recommend formal meditation practise (10-15 minutes) on non-course days, our adapted course also emphasised short ‘stat' practises lasting 30 to 90 seconds that could be engaged with during a clinical shift. These would include, for example, breath anchoring between patient appointments, mindful surgical-site preparation, and hand washing in the operating room.

***Supplementary Table 1. Week-by-week guide to the mindfulness course***

| **Week** | **Learning Outcome** | **Content** | **Techniques and self-practice** |
| --- | --- | --- | --- |
| **1** | **Complexities and challenges of today's healthcare environment and the impact on staff**    **Role of mindfulness and (self-)compassion tools for personal wellbeing and professional effectiveness** | Overview of course to build emotional intelligence competencies of self-awareness, self-management, social awareness & relationship management. | / |
| **2** | **Mindful self-awareness: becoming aware of our common habitual patterns when stressed** | Habitual patterns: autopilot, multitasking and mind- wandering, and understanding their impact on personal wellbeing and professional effectiveness in the clinical environment.    How mindfulness can help break our habitual patterns. | Anchor in the breath guided mindfulness practice    5 senses guided meditation practice |
| **3** | **Mindful self-awareness: thoughts** | How mindfulness can promote awareness of thoughts and thought patterns. | Thought observation guided mindfulness practice    Noting mental activity practice |
| **4** | **Mindfulness for self-awareness: emotions** | How mindfulness can help to promote awareness of emotions. | Body scan guided mindfulness practice    Noting physiological sensations practice |
| **5** | **Mindful self-awareness as a foundation to cultivate self-management through response flexibility** | Defining response flexibility.    Implementing response flexibility in practice with clinical applications. | SOS response guided meditation practice with examples in surgery |
| **6** | **Mindful self-awareness as a foundation to cultivate self-management through self-compassion** | Identifying common objections to self-compassion.    Understanding the elements of self-compassion.    Implementing self-compassion in practice with clinical applications. | Self-compassion guided meditation practice    Using a self-compassionate phrase or touch |
| **7** | **Using mindfulness to promote social awareness and relationship management** | Understanding the definition of, and barriers to, empathy and compassion and creating psychological safety.  Understanding why it’s important to shift from empathy fatigue into compassionate action to prevent burnout. | Empathy & compassion guided meditation practice    Mindful communication with clinical applications |
| **8** | **Mindfulness and gratitude** | Reflections and learnings from each participant. Sharing of personal and professional examples. | Gratitude journaling  Gratitude guided meditation practice |

## Supplementary Results

##

GLM models with the lowest Akaike Information Criterion (AIC) in RStudio are described below using patsy notation

### Attention

Formula = *scale(attention MAE) ~ Video + Intervention + (1 | participant)*

AIC = 147.3

McFadden R^2^ = 0.25

### Inattention

Formula = *scale(inattention MAE) ~ Video + Intervention + (1 | participant)*

AIC = 92.2

McFadden R^2^ = 0.72
